# Integrating virtual reality, electroencephalography, and transcranial magnetic stimulation to study the neural correlates of awe experiences: The SUBRAIN protocol

**DOI:** 10.1371/journal.pone.0302762

**Published:** 2025-04-02

**Authors:** Elena Bondi, Flavia Carbone, Marta Pizzolante, Giandomenico Schiena, Adele Ferro, Maddalena Mazzocut-Mis, Andrea Gaggioli, Alice Chirico, Paolo Brambilla, Eleonora Maggioni

**Affiliations:** 1 Department of Pathophysiology and Transplantation, Università degli Studi di Milano, Milan, Italy; 2 Department of Electronics, Information and Bioengineering, Politecnico di Milano, Milan, Italy; 3 Research Center in Communication Psychology (PsiCom), Università Cattolica del Sacro Cuore, Milan, Italy; 4 Department of Neurosciences and Mental Health, Fondazione IRCCS Ca’ Granda-Ospedale Maggiore Policlinico, Milan, Italy; 5 Department of Cultural Heritage and Environment, Università degli Studi di Milano, Milan, Italy; 6 IRCCS Istituto Auxologico Italiano, Milan, Italy; Mae Fah Luang University School of Anti Aging and Regenerative Medicine, THAILAND

## Abstract

**Introduction:**

Awe is a complex emotion unveiling a positive and mixed nature, which resembles the Romantic feeling of the Sublime. It has increasingly become the object of scientific investigation in the last twenty years. However, its underlying brain mechanisms are still unclear. To fully capture its nature in the lab, researchers have increasingly relied on virtual reality (VR) as an emotion-elicitation method, which can resemble even complex phenomena in a limited space. In this work, a multidisciplinary team proposed a novel experimental protocol integrating VR, electroencephalography (EEG), and transcranial magnetic stimulation (TMS) to investigate the brain mechanisms of this emotion.

**Methods:**

A group of bioengineers, psychologists, psychiatrists, and philosophers designed the SUBRAIN study, a single-center, one-arm, non-randomized interventional study to explore the neural processes underlying awe experiences. The study is ongoing and is expected to enroll fifty adults between 20 and 40 years of age. Currently, more than 40 individuals have been enrolled. The experimental protocol includes different steps: (i) screening, (ii) enrollment, (iii) pre-experimental assessment, (iv) VR experimental assessment, and (v) post-experimental debriefing. The brain’s electrical activity is recorded using the EEG while participants navigated three immersive awe-inducing VR environments and a neutral one. At the same time, the cortical excitability and connectivity is investigated by performing a TMS-EEG session right after each VR navigation. Along with cerebral signals, self-reported questionnaires were used to assess the VR-induced changes in the emotional state of the subjects. This data is then analyzed to delve into the cerebral mechanisms of awe.

**Discussion:**

This study protocol is the first one that tries to fully understand the neural bases of awe by eliciting and studying this phenomenon in VR. The pairing of awe-inducing VR experiences and questionnaires investigating participants’ affect and emotions, with non-invasive neural techniques, can provide a novel and extensive knowledge on this complex phenomenon. The protocol can inform on the combination of different instruments showing a reproducible and reliable setting for the investigation of induced complex emotions.

## Introduction

The sublime – the mixed feeling of pleasure and displeasure – has its roots at the heart of the eighteenth century, in a precise philosophical tradition that leads to Kant’s theory, according to which the sublime has a systematic-transcendental nature and is a uniquely subjective feeling [1–3]. This feeling-emotion is a combination of fear, enthusiasm, astonishment, and wonder [4], and has been linked with experiences of awe and terror [1]. Romanticism, furthermore, accentuates profound emotion as an authentic wellspring of aesthetic encounter [5]. It bestows fresh significance upon occurrences of wonder and awe by legitimizing such sentiments as reactions to the sublime in nature. In 2010, Richardson [6] establishes a connection between the eighteenth-century sublime (particularly that elaborated by Edmond Burke [1]) and neuroscience, while the following year, Onians [7] asserts that the profound awe of Burkean origin occurs due to the natural selection on our neural apparatus. In fact, Onians [7] suggests that our deep sense of awe stems from evolutionary developments in our brain. Over time, natural selection has fine-tuned our neural pathways to react strongly to vast or powerful experiences, as natural scenes, which make us feel overwhelmed and insignificant. Recently, the sublime and awe have increasingly become the subject of multidisciplinary and interdisciplinary investigations, combining philosophical, psychological, and physiological approaches [8–10]. The approach that we consider in the current research deems the sublime as a characteristic of specific stimuli able to elicit awe [11–13]. Specifically, a particular focus of psychology has been on awe as the subjective response to sublime stimuli [12,14], also besides the aesthetic domain [15,16]. Therefore, while the sublime could be considered as an inherent property of a stimulus, awe is its emotional correlate. Despite initial neural evidence on the sublime stimuli [17], the understanding of awe’s underlying brain mechanisms remains at an early stage. Alike the sublime [1], awe emerged as an intense emotional state combining positive and negative components simultaneously [12,18], which arises in response to stimuli perceived as perceptually (e.g., waterfalls, trees, mountains, and tempests) [16] or conceptually [19] vast or overwhelming. From a psychological level, awe has been correlated with a reduction on aggressive behaviors [20] an increase of attentional focus [21], and an extension of time perception [22]. Moreover, studies showed that awe seemed to be involved in the reduction of rumination and self-referential thinking [23–27], which are factors characterizing mood disorders, such as Major Depressive Disorder (MDD). Most studies have used awe-inducing videos or images to elicit awe in the lab, but these tend to elicit more subtle emotions with respect to those evoked by real-life experiences. In this context, virtual reality (VR) technology can be used to create a more intense laboratory version of awe [16,28]. VR is capable of creating realistic scenarios [28] by also providing a multisensory stimulation. Compared to other emotion-inducing tools, VR provides a unique sense of presence and immersion and a heightened emotional intensity of the experience [29]. In 2018, our research team already validated three awe-inducing immersive VR environments representing natural scenarios (i.e., forest, mountains, and Earth view from deep space) [30], since nature is recognized as the most frequent trigger of awe-inspiring experiences [31], as compared to a non-awe-inducing natural environment with green grass, trees, and flowers.

Although the mechanisms underpinning awe experiences remain largely elusive, awe-inducing VR scenarios designed for a laboratory setting can allow us to identify experiential, behavioral, cognitive, and brain correlates of awe. So far, few studies have investigated the brain activity correlates of the awe emotion through functional Magnetic Resonance Imaging (fMRI). In 2014, Ishizu and Seki report the results of a study conducted on 21 subjects to whom images of natural beauty and natural sublimity were shown while their brains were examined using fMRI technology [17]. Ishizu and Seki’s studies demonstrate that natural beauty and natural sublimity activate different brain areas and evoke very different experiences in humans [17,32,33]. Specifically, the medial orbito-frontal cortex [32,33], sensorimotor regions, dorsolateral prefrontal cortex (DLPFC), and insula [32] were found correlated with natural beauty, whereas the inferior temporal cortex, hippocampus, inferior/middle frontal gyri, basal ganglia, and cerebellum were suggested as modulators of the intensity of sublimity [17]. Finally, few fMRI studies investigating the awe experience suggested the involvement of the fronto-parietal network, including medial frontal gyrus, insula, and supramarginal gyrus, and the reduction of the default mode network (DMN) within awe experiences [24] and the involvement of left middle temporal gyrus as pivotal in processing both positive and threatening awe experiences and explored its interactions with other brain regions usually involved in emotional processing [34]. Despite this preliminary fMRI evidence, a more profound comprehension of the neural underpinnings of the awe experience is needed. In this context, a deeper knowledge of the brain activity and connectivity patterns underlying this complex experience can be obtained by electroencephalography (EEG) and its combination with transcranial magnetic stimulation (TMS). TMS and EEG are non-invasive techniques that provide direct insights into brain networks’ instantaneous and directional interactions [35–38]. Unlike fMRI, the EEG technique provides direct information on the brain’s electrical activity [39], boasting exceptional time resolution to explore functional brain connectivity on the neuronal time scale across multiple frequency bands [35,38], while TMS enhances the EEG potential for studying the propagation of information within the brain networks [36,37]. By leveraging EEG to track TMS pulses through functionally linked brain regions, TMS-EEG integration emerges as a valuable tool to investigate awe-induced biomarkers, such as changes in the cortical excitability and effective (directional) connectivity. Hence, TMS-EEG integration holds promise in unveiling how the propagation of neural information within the brain networks is affected by sublime emotional experiences elicited by means of nature-based VR scenarios. Previous studies investigated the neural correlates of awe using images, clips or mixed-reality [40–43], whereas only a recent one [44] used VR to simulate a rocket launch, overall showing the involvement of power changes in theta and beta bands. Most of these studies focused on the space environment, by assessing the neural correlates of awe induced by that specific scenario [40,41,43,44], whereas Hu et al., [42] did not specify the clips used to investigate awe correlates.

Within this context, for the first time, the multidisciplinary SUBRAIN project investigates the psychological and neural correlates of different laboratory instances of awe, with the ultimate goal of gaining a complete picture of the cerebral building blocks of this complex emotional experience. This ambitious aim is reached via the development of a new multimodal research protocol that integrates several immersive nature-based VR scenarios, clinical questionnaires, EEG, and TMS. Building upon our previous pilot study [10], which explored the potential of EEG to detect neural variations during VR navigation scenarios, the present research extends this methodological approach. We propose a novel research protocol designed to comprehensively investigate distinct instances of awe through an integrated multimodal neuroimaging and emotional assessment framework. Specifically, our methodology incorporates simultaneous EEG recording, TMS-EEG, and validated emotional questionnaires to provide a broad examination of neural correlates and subjective emotional experiences to awe. The primary technical challenge was integrating the VR oculus system with the TMS stimulation protocol, specifically the selection of the stimulation target point. As formerly reported, only a few studies investigated the brain correlates of awe, finding them in the middle frontal cortex, which includes the DLPFC, temporal regions, and DMN [24,34]. Moreover, from a psychological perspective, awe seems to reduce the ruminative and self-referential thinking [23–27], which are commonly associated with MDD. Awe could promote changes potentially contrasting the biological signatures of MDD and the phenomenology of MDD experience reducing rumination and hopelessness [26,27]. However, considering the limited knowledge regarding the impact of awe on MDD, a potential expansion of this study could involve investigating the neurobiological correlates of awe in individuals with MDD, which had widely shown lower activation of the left DLPFC [45–47] and decreased deactivation in DMN [48] with respect to healthy individuals. Besides this aspect, our research protocol aims to combine TMS-EEG and the VR cap, which leads to some limitations in the definition of the stimulation target point because of the physical interaction between the TMS coil and the VR cap. Considering the regions seemingly involved in the awe processing, the regions involved in MDD symptomatology, the TMS stimulation properties [49], and the coverage of the VR headset that limited the ability to stimulate the DMN and temporal regions, the left DLPFC has been chosen as the target point for the TMS-EEG protocol. Besides the suggested involvement in awe experiences, the DLPFC was also found correlated with valence of emotional pictures [50] and playing a central role in emotion regulation processes [51]. Therefore, the choice of using the DLPFC as stimulation target to investigate TMS-EEG biomarkers of the awe experience became natural.

The present protocol adopts a multidisciplinary approach, with the goal of investigating the brain activity and connectivity changes induced by awe in a laboratory setting, by exploiting physiological – EEG and TMS-EEG – and psychological – emotional questionnaires – measures. Based on former awe literature [24,34], we hypothesized an effect of awe, induced by validated awe-inducing VR scenarios [30] compared to non-inducing ones, primarily in the frontal and temporal regions. In our study, these changes are investigated by analyzing the EEG signals recorded when participants navigate inside awe-inducing VR scenarios and by exploiting single-pulse TMS-EEG over the left DLPFC, part of the frontal lobe believed to be affected by awe [24]. Furthermore, we hypothesized that by stimulating the DLPFC, we could assess changes, induced by awe, in the cortical excitability and connectivity in the target and connected regions. Therefore, we expect to identify possible EEG and TMS-EEG biomarkers related to awe, by comparing EEG and TMS-EEG measures in awe-inducing VR scenarios with respect to non-inducing VR scenario and baseline ones. Finally, we hypothesize that this innovative multimodal approach can provide key evidence of the psychological and neural correlates of awe experiences, which paves the way to plan experiential interventions for the promotion of mental well-being.

## Methods

### Study aims

The SUBRAIN study was born out of a multidisciplinary collaboration between biomedical engineers, psychologists, psychiatrists, and philosophers. It aims to provide unprecedented knowledge on the physiological, emotional, and neural mechanisms underlying the awe emotion. The following specific aims are pursued: (i) to test the feasibility of a multimodal neuro-centered experimental approach for studying the neurobiological bases of the awe experience in a laboratory environment, (ii) to explore brain responses to different VR experiences of awe, and (iii) to identify brain mechanisms linked with type and intensity of awe. The research protocol has been registered with Open Science Framework (https://osf.io/m47du).

### Study population

Fifty young adults from the general Italian population will take part in the study. Recruitment started on February 18^th^, 2022, and is currently ongoing. The sample recruitment is performed via word of mouth. Inclusion criteria are (i) 20-40 years of age, (ii) normal or corrected vision, and (iii) capability to give informed consent and to comply with the study procedures. Exclusion criteria are (i) balance or vestibular disorders, (ii) severe migraine/headache, (iii) pregnancy, (iv) metal/electric implants in the head-neck district, (v) severe cognitive deficits, (vi) medical conditions predisposing to greater epileptic risk or side effects during TMS (including personal or family history of epilepsy, brain ischemic events, neurological diseases, neurosurgical interventions, vascular or orthopedic head-neck surgery, major head trauma, severe migraine or headaches), (vii) heart, respiratory, kidney, liver failures or immuno-suppression, (viii) substance use disorders, (ix) history of psychotic or mood disorders based on DSM-5, and (x) intellectual disability. Participation in the study is conditional on the signature of the written informed consent to the study protocol and fulfillment of the eligibility criteria.

### Study design

**Fig 1:**
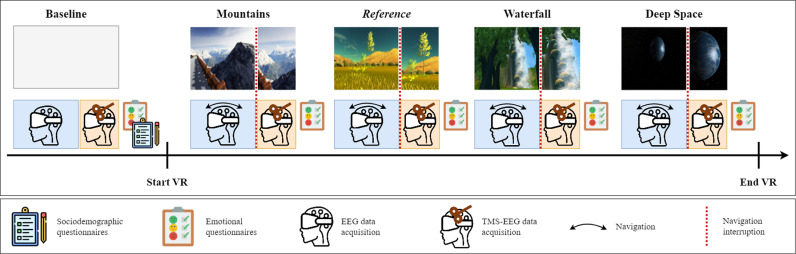
Experimental protocol. EEG and TMS-EEG data are recorded at baseline, along with sociodemographic and emotional questionnaires. EEG data is recorded during each VR scenario and TMS-EEG is recorded right after the end of the navigation. Afterward, emotional questionnaires are administered. VR scenarios are administered in a Latin Square counterbalanced order.

This is an Italian, single-center, one-arm, non-randomized interventional study. The study procedure involves four main phases, (i) screening and enrolment, (ii) pre-experimental assessment, (iii) VR experimental assessment, and (iv) post-experimental debriefing, which are schematized in Fig 1 and detailed in the homonymous subsections of “Interventions”.

The study protocol conforms to the Helsinki Declaration and has been approved January 26^th^, 2021, by the competent Ethical Committee of the Fondazione IRCCS Ca’ Granda Ospedale Maggiore Policlinico, Milan (OSMAMI-26/01/2021-0002688-U).

### Study material

#### Questionnaires.

Information regarding the participants general information, such as biographic, socio-demographic, and clinical information, along with emotional status during the entire experiment, are needed to evaluate the compliance of the participants to the inclusion criteria and the subjective effect of the awe experience. Currently, we are employing the following questionnaires:

1. Case Report Form (CRF): ad-hoc questionnaire for collection of biographic, socio-demographic, and anamnestic information, administered to aspirant participants to check their fulfillment of the inclusion criteria and to investigate any previous experiences with VR environments.
2. Short Positive And Negative Affect Scale (PANAS): a brief version of the homonymous scale that measures two main clusters of affective experiences, positive affect and negative affect, by rating 20 adjectives on a 5-point Likert scale [52].
3. Single Item Emotion (SIE) scale: a questionnaire that rates eight emotions, including awe, on a 7-point Likert scale [30].
4. Short Awe Scale (SAS): brief 7-item version of the Awe-Experience Scale [41], which explores the intensity of perceived awe [53].
5. Hamilton Depression Rating Scale (HDRS): a 17-item measure of depression severity rated by the clinician [54].
6. Visual Analog Mood Scale (VAS): a single-item measure of feeling state rated by the participant [55].

#### VR equipment and scenarios.

The study of the complex awe emotion in a laboratory setting needs specific controlled laboratory conditions [28,30]. VR is a technology that uses multi-sensory stimuli to create the perception of being “present” in computer-generated environments, making it the preferred technology to investigate awe experiences. To provide a multi-sensorial environment, the VR equipment should permit immersive and interactive experiences, whereas the VR scenarios should be built and validated to induce awe emotions. As control condition, a non-inducing VR scenario should be included in the protocol, to control the awe effect.

The currently used VR system is composed of an Oculus Rift DK2 (Oculus VR LLC, Irvine, CA, United States) equipped with headphones and a Microsoft Xbox controller and connected to a computer with an NVIDIA GTX1070 graphic card and Intel i7 GPU. The Oculus Rift DK2 is a head-mounted display with a 1920 × 1080-pixel resolution and 90 Hz frame rate. Four interactive and immersive VR scenarios modeled using the Unity software (version 5.5.1) are employed. All scenarios represent natural scenes; three of them have been validated to induce different instances of awe, whereas the remaining one is a neutral, reference condition [30]. The three awe-inducing scenarios represent natural scenes of (i) a forest with tall trees, (ii) high mountains, and (iii) the Earth viewed from deep space. The neutral reference scenario represents a green grass with few flowers and trees. In the (i) and (ii) scenarios, environmental sounds consistent with the virtual landscapes are added to the visual stimuli with an intensity proportional to the participant’s proximity to the sound source. The scenarios can be visualized at https://www.youtube.com/watch?v=KiOwpVviK74.

#### EEG and TMS-EEG equipment.

The investigation of neural correlates of the awe emotion during the navigation, using only EEG, and after the navigation, by pairing single-pulse TMS and EEG, should be based on high-density TMS-compatible EEG cap, with at least 64 channels, with a sampling frequency ideally of 5 kHz. Moreover, the TMS device should be able to deliver a focal stimulation on the target location and be paired with a neuronavigation system to allow reproducibility and consistency between the different experimental conditions.

The currently used equipment is composed of a Geodesic EEG system 400 (GES 400, Electrical Geodesic Inc., Philips, the Netherlands), with sampling frequency of 1kHz, equipped with a TMS-compatible 64-channel MicrocelGeodesic sensor net (GSN 100, Electrical Geodesic Inc., Philips, the Netherlands). The EEG device is combined with an STM9000 TMS system (Ates Medica Device s.r.l, Italy) equipped with a 70-mm butterfly cooled coil and with the NetBrain 9000 neuronavigation system (EBNeuro S.p.A, Italy).

### Interventions

The four phases of the study procedure are described in the following dedicated sections.

#### Screening and enrolment.

In this phase, the aspirant participant is informed of the study procedures and freely decides whether to consent to participate in the study. If so, after the signature of the written informed consent, a univocal code is assigned to the aspirant participant. Eligibility criteria and previous experiences with VR are investigated via the administration of the CRF questionnaire. If inclusion criteria are met, the participant is invited to a second visit that includes the three remaining phases.

#### Pre-experimental assessment.

The participant’s emotional status is retrieved via the short PANAS, SIE, and SAS questionnaires. The participant’s mood is explored via the HDRS and VAS questionnaires. The experimental setup is prepared through the montage and functional check of the VR, TMS, and EEG devices (see S1 Fig for the setup). Eyes-closed resting-state EEG recording (lasting 2 minutes) and TMS-EEG recording (lasting about 3.5 minutes) are performed. More details can be found in the “TMS-EEG protocol” section.

#### VR experimental assessment.

After receiving instructions about the VR experiment, the participant navigates the VR scenarios (three awe-inducing and one of reference) in a counterbalanced order, defined using the Latin Square design, for about 3 minutes each. Before each experience, the participant is instructed on how to navigate and is asked to keep her/his eyes closed until the scenario has started. EEG signals are continuously recorded during navigation in each VR scenario. An event marker is set on the EEG signal when the participant opens the eyes and starts the navigation. In the awe-inducing scenarios, a further marker is set when the scene with hypothesized maximum awe intensity is reached, after which the navigation continues for at least 30 seconds. Right after, the participant is requested to stay still, looking at the VR-induced landscape in front of her/him, while TMS-EEG recordings are performed (lasting about 3.5 minutes, see “TMS-EEG protocol”). After the TMS-EEG session, the participant’s emotional and mood status is assessed through the re-administration of the short PANAS, SIE, SAS, and VAS scales. The maximum intensity of awe is thought to occur when something unexpected happens. The unexpected can also be defined as an operationalization of the need for accommodation [28], an essential ingredient, when combined with vastness, to evoke an intense response of awe. The awe-inducing VR scenarios employed in this study [30] were built creating a standardized navigation path conducing to an unexpected cue scene, i.e., for the forest environment, it was the view of the waterfall, for the high mountains, it was the view of the wild panorama and the cliff, for the Earth from the space environment, it was getting as close as possible to the Earth itself, right outside its atmosphere.

#### Post-experimental debriefing.

At the end of the VR experiment, main participant’s perceptions and feelings concerning the VR experiences are captured through a debriefing with the psychologist, which is recorded upon the participant’s consent.

### TMS-EEG protocol

The TMS-EEG protocol should be defined considering the following parameters: (i) the unknown duration of the awe emotion, after the scene with hypothesized maximum awe intensity is reached, (ii) the total length of the protocol to reduce the nausea state as much as possible, and (iii) number of TMS stimuli needed to obtain a reliable response, e.g., a clear TMS-evoked potential (TEP).

Considering that the duration of the awe feeling is still unknown, the participants can undergo the TMS-EEG session shortly after reaching the awe peak and right after ending the navigation while still immersed in VR scenarios. By doing this, the TMS-EEG could capture either instantaneous or short-term effects of the awe peak. In the designed protocol, 30 seconds after reaching the awe peak, the participants are asked to stop navigate, stay still, and keep looking at the scene of the VR scenario in front of them while being stimulated with 105 TMS pulses over the left DLPFC at 0.5 Hz with a stimulus intensity around 120% of the individual resting motor threshold (rMT). To reduce habituation and expectation effects [56], the stimulation frequency should be jittering. Of note, the chosen stimulation frequency allows for the extraction of both an adequate pre-stimulus window (of at least 1 second) and the TEPs of interest [57]. The chosen stimulation site, i.e., the DLPFC, allows for measuring the impact of the VR scenarios on a region that is known to contribute to affective processing and seems to be specifically involved in awe experiences [24,34]. In the pre-experimental phase, the rMT is measured as the minimum intensity needed to obtain 5 out of 10 consecutive right-index movements when stimulated by the left primary motor cortex. In a desired configuration, the rMT should rely on the observation of motor evoked potentials of at least 50 uV in the relaxed right first dorsal interosseous [58]. The stimulation site of the DLPFC is then defined as an electrode-free point using as reference the EEG cap electrode E12 (F3), around 5 cm ahead of the stimulation motor point. The coil is placed tangentially to the scalp and perpendicular to the EEG wires’ direction in order to reduce the TMS-induced decay artifact [59,60]. To ensure that the stimulation point is inside the DLPFC and to increase the intra-subject reproducibility of the stimulation across different TMS-EEG sessions, the neuronavigation system is used. Through preliminary tests, the stimulation point location and intensity are chosen considering the TMS-related artifacts on the EEG, the comfort of the patient, and using as anatomical reference the middle frontal gyrus defined using the Atlas69 atlas on a template image of the neuronavigator (S2 Fig). According to the listed parameters, the stimulation point location and coil orientation are saved in the neuronavigator and replicated in the following TMS-EEG sessions. Lastly, to reduce the auditory evoked potential evoked by the TMS pulses, a white noise signal is reproduced during the TMS-EEG sessions through the headphones of the Oculus Rift DK2. The volume of the white noise is adjusted for every participant to cover the TMS clicking noise and accommodate their comfort.

### Sample size calculation

The SUBRAIN study was designed as purely explorative, without any hypotheses on the size of the physiological behavioral and brain responses to the innovative VR protocol. For these reasons, the sample size calculation was originally based on the financial resources available for the study conduction. Nevertheless, a hypothetical power calculation – considering as outcome the identification of EEG theta power changes in the DLPFC – has been done. We found that our sample of 50 subjects should enable us to detect a 0.2 mean difference (0.4 standard deviation) in the relative power spectral density in awe-inducing vs. reference VR using a paired t-test (p < 0.05) with 93% of power. The outcome of the proposed experimental protocol will also be measured in terms of the possibility of (i) effectively sending the TMS pulses to the target region, taking into account the VR equipment, (ii) obtaining good quality EEG signal during the VR protocol to extract features of interest, and (iii) effectively removing TMS-induced artifacts from the EEG signals. Considering the ISO guidelines of the technical committee ISO/TC 210 (“Quality management and corresponding general aspects for medical devices”) regarding usability testing of medical devices, the use of the new experimental protocol on 5/10 subjects should have allowed most ( > 70%) of the technical problems of integration of the three systems (VR, EEG, and TMS) to emerge. These problems have been examined in the pilot sample (n = 6) and addressed to refine the experimental protocol, which is being applied to the rest of the study sample. Finally, the study’s statistical power, in terms of emotional-behavioral and other cerebral correlates of experiences in VR will be further examined and discussed retrospectively.

### Data management plan

Experimenters guarantee the anonymity of the volunteers, treating their common and sensitive data, excluding the personal ones, and avoiding their association with the person by using a pseudonymized form. At the beginning of the experiment, the experimenters associate a code that identifies the subject’s data during all the experimentation phases. The experimental data, including signals, socio-demographical and anamnestic data, and questionnaires, are recorded, analyzed, and preserved along with the code. The file associating participants’ codes and their identification data and the one containing experimental data associated with the participants’ codes are stored on a password-protected computer. Only experimenters and authorized personnel can link the code to the name of the volunteer. Data is recorded, processed, and stored by electronic and non-electronic devices and will be disseminated only in strictly pseudonymized form, such as through scientific publications and conferences.

### Primary endpoints

Development of the experimental and innovative setup based on the successful combination of VR, TMS, EEG, and clinical questionnaires to induce different instances of the awe experience and study its emotional-behavioral and cerebral correlates. Particularly, (i) setup, test, and refinement in a pilot sample (n = 6), and usage in an independent sample (n = 44), and (ii) identification of emotional-behavioral and brain functional mechanisms, including neuronal oscillatory activity, cortical excitability, and pathways of propagation of neuronal information, underlying the awe experience.

### Secondary endpoints

Identification of specific emotional-behavioral and brain functional mechanisms underlying the different VR-induced awe experiences. Specifically, investigation of (i) the different types and intensities of awe inspired by VR scenarios, (ii) the neural correlates of the subjective awe experience, reported in the questionnaires, independently of the VR scenario itself, and (iii) the specific neural correlates of each VR scenario, by means of comparison between the sublime-inducing VR scenario and the reference one.

### Safety considerations

The project does not involve administering drugs or other substances or invasive clinical practices. Therefore, no adverse events are expected. The interventional design of the study is exclusively due to the use of the innovative VR-TMS-EEG experimental setup, whose components are non-invasive. TMS stimuli, when administered repetitively, several times per second, can induce seizures in predisposed subjects (0.003% of cases). Such seizures are of short duration and leave no consequences. The protocol involves sending TMS stimuli at intervals of more than one second apart. In addition, personal or family history of epilepsy, neurological diseases, neurosurgical interventions, and major head trauma that increase epileptic risk constitute exclusion criteria for the study. At stimulation frequencies such as those used in our study, the risk of seizure induction in subjects not predisposed to develop seizures is negligible (less than 2 cases per 1000 stimulation sessions). The prolonged use of VR can produce discomfort, such as nausea, dizziness, and increased muscle fatigue.

### Statistical analyses

EEG and TMS-EEG data pre-processing is carried out using BrainVision Analyzer (BrainProducts, Germany) and EEGLAB, an open-source toolbox running in Matlab R2022a (Mathworks, Inc., Natick, Massachusetts, USA). Neuronal and emotional questionnaire data are analyzed using in-house Matlab scripts.

Statistical data analyses aim to evaluate the differences, in terms of emotional questionnaire scores and neural correlates, induced by (i) the experience of awe, comparing the awe-inducing VR scenarios with the reference VR one and (ii) the VR, comparing the reference VR scenario with the baseline. A further objective is to correlate the neuronal changes with the emotional ones. To achieve these goals, (i) the values of intensity of emotional dimensions recorded with the SAS, SIE, and PANAS questionnaires are compared using paired t-tests or Friedman statistics across conditions to evaluate the subjective effect of the different VR scenarios. Brain complexity, power, and functional and effective connectivity features are extracted from the EEG signal and used to evaluate differences of multiple cerebral dimensions among the experimental conditions, ultimately providing a wide comprehension of the neural bases of the awe emotion. Amplitude and latency of TEPs of interest [61] and TMS-evoked effective connectivity indices [62] are calculated from the TMS-EEG data and compared among the experimental conditions. Power and connectivity analyses are performed in the main EEG frequency bands (delta: 1-4 Hz, theta: 4–8 Hz, alpha: 8–13 Hz, beta: 13–30 Hz, and gamma: 30–50 Hz), excluding from the TMS-EEG analyses the delta band, whose activity might be influenced by the stimulation frequency. Multi-scale connectivity features are extracted using graph theory. Finally, the association between EEG and TMS-EEG features and scores in the emotional questionnaires is further investigated via generalized linear models.

### Current status

The setup, test, and refinement of the experimental protocol, which is a primary endpoint of the SUBRAIN study, have been achieved by successfully combining TMS, EEG, VR and by refining their integrated usage in the experimental session. Up to now data from forty-two participants (18M, 24F, 26.40 ±  4.16 years), including six participants for the pilot study, have been recorded. Using the proposed protocol, a pilot study on six healthy controls showed the possibility of extracting standard TEPs, in line with the literature, when stimulating the left DLPFC at baseline [61], ensuring the reliability and reproducibility of the protocol even with suboptimal instrumentations. This pilot study has been followed by the inclusion of the neuronavigator in the experimental equipment, which has allowed us to improve the intra-subject reproducibility of TMS pulses sent after the navigation in different VR scenarios. Also, to ensure the robustness of the analyses, the nauseous state of the subject, found especially after the reference scenario, was tracked with annotations with the goal of incorporating it into statistical models. The endpoints relative to the investigation of the psychological and brain correlates of the VR-induced awe experience are still under study.

## Discussion

Through the incorporation of pioneering quantitative, multidisciplinary, and multimodal experimental and analytical methodology, this protocol aims to enhance the currently limited understanding of the cerebral foundations of the awe emotion. We seek to present fresh insights into the collaboration of various brain regions in generating the profound, enigmatic, and transformative experience of awe. This protocol employs a meticulously designed framework that integrates VR with neural techniques, including EEG and TMS, alongside emotional questionnaires, to investigate the mechanisms underpinning VR-induced awe experiences.

The preliminary data obtained so far demonstrates the proposed protocol’s feasibility and reproducibility, unveiling distinctions of neural mechanisms across various VR-induced awe scenarios. The use of interactive and immersive VR as an awe-inducing medium enables the participant’s full and ecological immersion in the experience, as previously demonstrated [28,30], whereas the combination of EEG and TMS enables the extensive exploration of the cortical changes induced by diverse instances of awe through VR scenarios, offering novel insights into this intricate emotional experience. Integrating different neural techniques and tailored psychological questionnaires contributes to unveiling the specific emotion under study and opens the door to new insights into the brain mechanisms underlying the perception of complex emotions.

A central question arising from this investigation pertains to the potential use of experiential interventions based on the elicitation of awe emotion to promote mental health. At a biological level, preliminary findings suggest awe to decrease DMN activity [24], sympathetic activation [18], and pro-inflammatory cytokines [63]. Moreover, awe seems to reduce the ruminative and self-referential thinking [25–27], which are known to characterize some psychiatric disorders, such as MDD. Considering the lacking knowledge of the impact of awe in MDD, a possible extension of the proposed study will employ this experimental framework in the study of MDD to gain a deeper understanding of the neurobiological correlates of awe in affected individuals, paving the way for testing its potential in complementing traditional therapies for MDD.

While focusing on the awe emotion, our approach offers a reproducible framework adaptable to diverse research domains, especially those exploring immersive experiences. However, several limitations merit consideration. Given the recent interest in the study of the neuronal activity underlying the complex awe emotion and the limited studies on the phenomenon itself, few open questions remain. Firstly, the duration of the awe feeling is still unknown, leading to difficulty in defining the precise timings for the investigation of the neural correlates of the awe peak. For this reason, the proposed protocol exploits both EEG during navigation, which capture the neural dynamics associated with the awe peak, and TMS-EEG recordings shortly after the awe peak, which might capture either the instantaneous or the short-term effects of awe. The awe-inducing VR scenarios used in this study have been previously validated [30] for awe and have been built by creating a surprise scene that operationalizes the need for accommodation [28] that occurs with the maximum peak of awe intensity. However, given the limited knowledge on neural correlates of awe, a pilot study could be included to evaluate the synchronicity of dynamic awe-related features and the scenarios’ structure. Moreover, some studies reported discrepancies between physiological responses and reported feelings [9,64]; despite the lack of strong hypotheses on the EEG correlates of perceived awe, this possibility needs to be taken into consideration.

Furthermore, open questions on any effects of low frequency TMS on the investigated intervention (awe induction via VR) remain. In this study, TMS was used as an exploration tool and not as an intervention, but we cannot exclude any interactions between the TMS stimulation and the intervention itself. This interaction could be studied in the future, evaluating a possible interaction between stimulation and awe feeling by stimulating different awe related regions, non-awe related regions as control condition, and sham stimulation. Another limitation, regarding the mean by which awe is induced is that VR could induce sickness and nausea in some patients [65] which could, in turn, impact the experience, altering the experience of awe and its beneficial effects. However, in our protocol, the nausea can be considered an invariant condition during the VR navigation not affecting the evaluation of the awe effect on the brain, which is based on the relative statistical comparison of the awe-inducing VR scenarios with the reference one. A questionnaire evaluating the level of nausea after each VR scenario should be implemented to keep track of this phenomenon. Our study implemented three different awe-inducing scenarios designed to enhance the vastness dimension [19,31], however, other awe dimensions could be investigated, increasing the number of possible new VR scenarios. Finally, the use of EEG in this protocol provides valuable insights into cortical activity during awe experiences but poses intrinsic limitations when investigating activity in deep brain regions, such as the DMN, due to its low spatial resolution. While combining EEG with TMS offers an opportunity to probe cortical excitability and connectivity, it remains challenging to infer precise activity changes in subcortical or deeper cortical structures. The use of higher spatial resolution techniques, such as fMRI, could be used to infer alterations in deep brain regions. Nevertheless, the possibility to combine EEG with VR and TMS makes this technique the most suitable for our research purpose, that is, to understand the functional brain correlates of the awe emotion elicited in an immersive environment.

## Conclusions

Our study results from an unprecedented synergistic integration of complementary expertise in bioengineering, psychology, philosophy, and psychiatry. SUBRAIN adopts an innovative, quantitative, and multimodal experimental and analytical approach to increase the currently limited knowledge of the cerebral bases of the awe experience. This protocol not only provides new evidence on how multiple brain areas can work together to produce the powerful, mysterious, transformative awe emotion, but also sets the methodological precedent for future experiments in affective neuroscience. The integration of immersive VR with EEG and TMS-EEG recording offers a robust framework for investigating the neural underpinnings of complex emotions in ecologically valid settings.

## Supporting information

S1 Fig Experimental setting.
The photograph shows the experimental setting during the navigation in a VR scenario, while recording the EEG signal.
(TIF)

S2 Fig Projection on the scalp of the TMS stimulation target point.
The stimulation site over the left dorsolateral prefrontal cortex is identified by referring to the F3 electrode of the EEG cap placed over the scalp of the participant. The middle frontal gyrus (red) defined using the Atlas69 atlas of the neuronavigator is also used as anatomical reference.
(TIF)
