# Supplementary figures and images for: Integrating virtual reality, electroencephalography, and transcranial magnetic stimulation to study the neural correlates of awe experiences: The SUBRAIN protocol
Source: PLoS One. 2025 Apr 2;20(4):e0302762. doi: 10.1371/journal.pone.0302762 (PMC11964456; doi:10.1371/journal.pone.0302762)

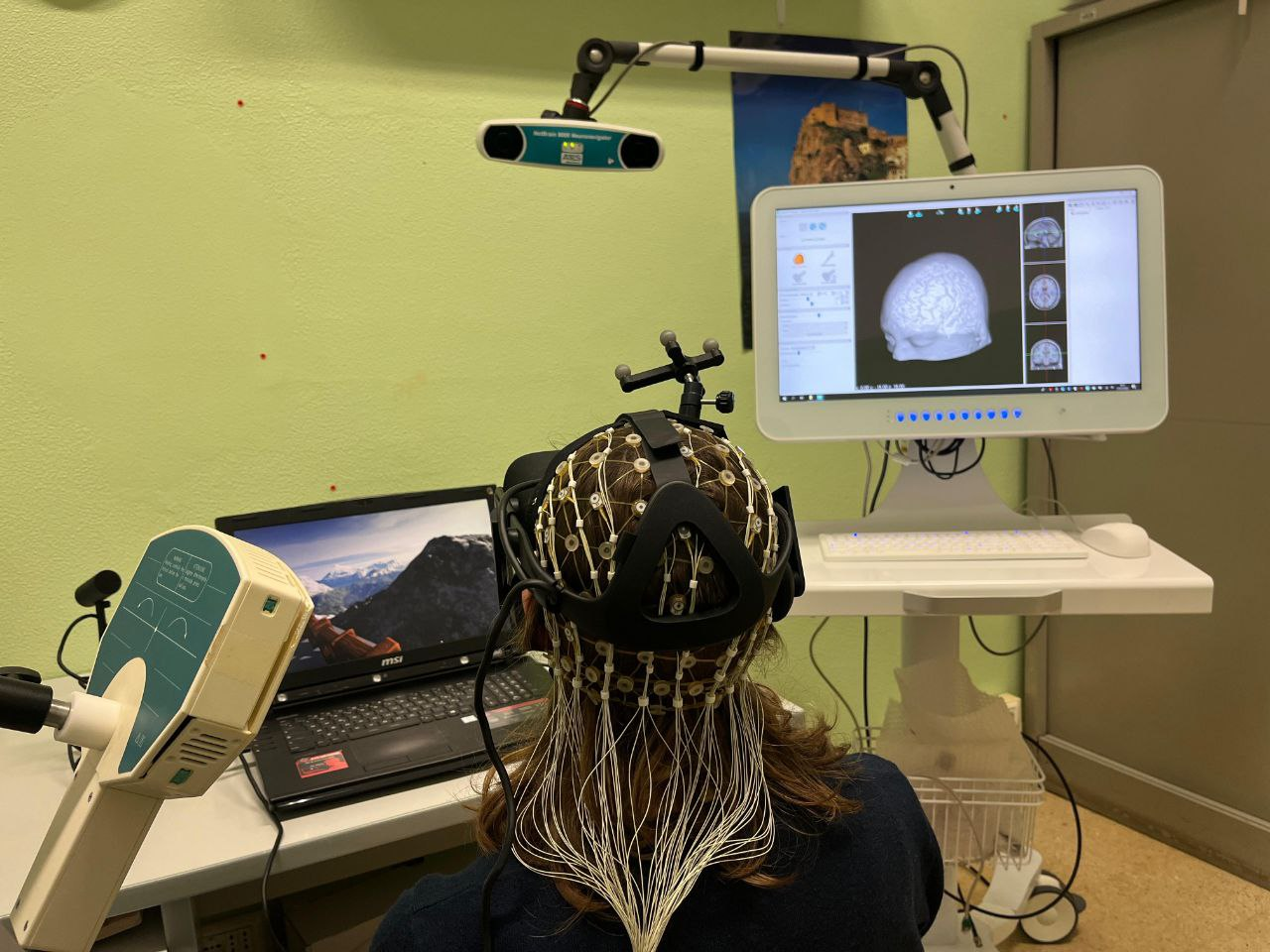

Supplement: S1 Fig — The photograph shows the experimental setting during the navigation in a VR scenario, while recording the EEG signal. (TIF) [file pone.0302762.s001.tif]

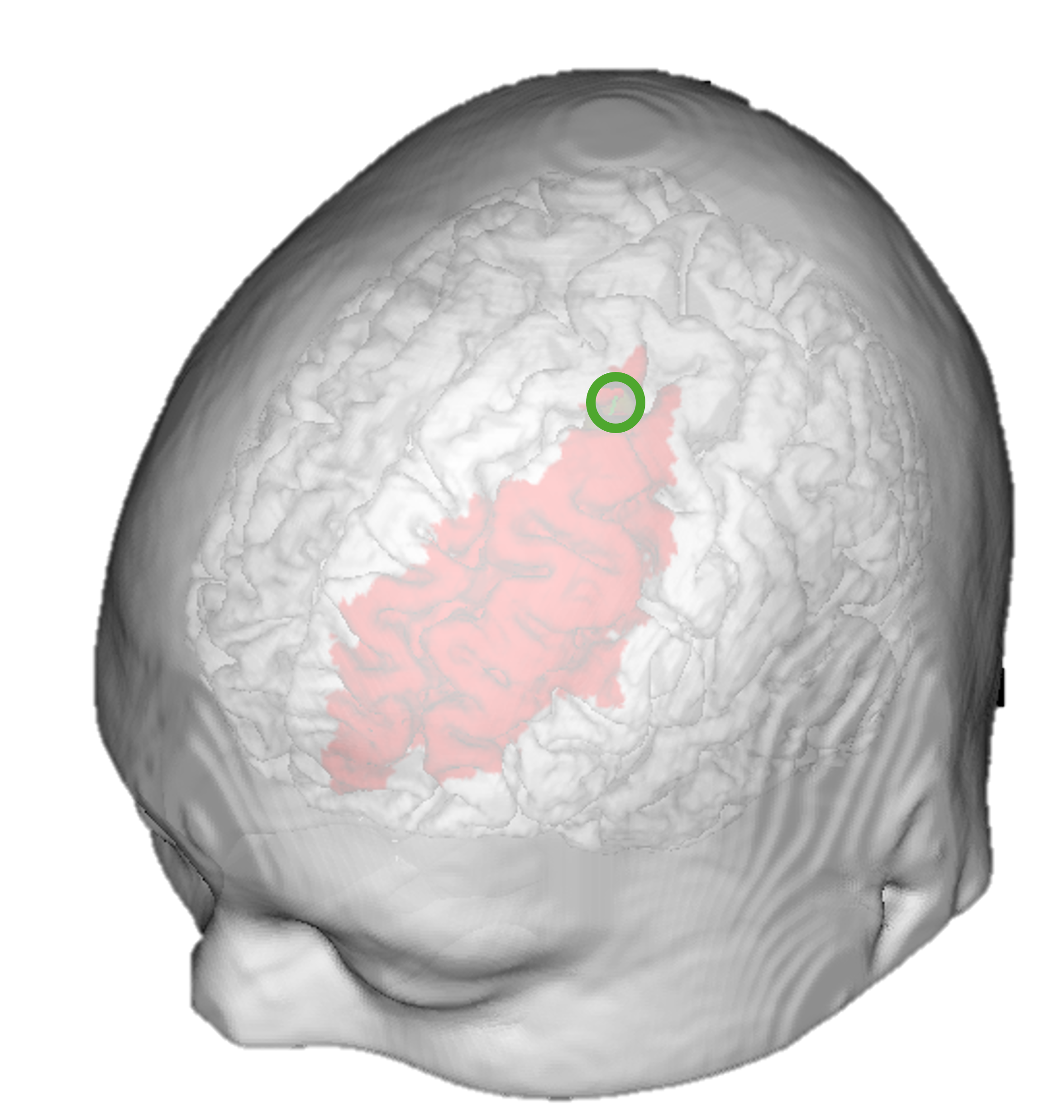

Supplement: S2 Fig — The stimulation site over the left dorsolateral prefrontal cortex is identified by referring to the F3 electrode of the EEG cap placed over the scalp of the participant. The middle frontal gyrus (red) defined using the Atlas69 atlas of the neuronavigator is also used as anatomical reference. (TIF) [file pone.0302762.s002.tif]
